# Angstrom Thick ZnO Passivation Layer to Improve the Photoelectrochemical Water Splitting Performance of a TiO_2_ Nanowire Photoanode: The Role of Deposition Temperature

**DOI:** 10.1038/s41598-018-34248-3

**Published:** 2018-11-05

**Authors:** Amir Ghobadi, Turkan Gamze Ulusoy Ghobadi, Ferdi Karadas, Ekmel Ozbay

**Affiliations:** 1 UNAM–National Nanotechnology Research Center, Bilkent University, Ankara, 06800 Turkey; 2 Institute of Materials Science and Nanotechnology, Bilkent University, Ankara, 06800 Turkey; 3 NANOTAM - Nanotechnology Research Center, Bilkent University, Ankara, 06800 Turkey; 4 Department of Electrical and Electronics Engineering, Bilkent University, Ankara, 06800 Turkey; 5 Department of Energy Engineering, Faculty of Engineering, Ankara University, Ankara, 06830 Turkey; 6 Department of Chemistry, Bilkent University, Ankara, 06800 Turkey; 7 Department of Physics, Bilkent University, Ankara, 06800 Turkey

## Abstract

In this paper, we demonstrate that angstrom thick single atomic layer deposited (ALD) ZnO passivation can significantly improve the photoelectrochemical (PEC) activity of hydrothermally grown TiO_2_ NWs. It is found that this ultrathin ZnO coating can passivate the TiO_2_ surface defect states without hampering the carrier’s transfer dynamics. Moreover, a substantial improvement can be acquired by changing the deposition temperature of the ZnO layer (80 °C, and 250 °C) and named as 80 °C TiO_2_-ZnO, and 250 °C TiO_2_-ZnO. It was found that the deposition of this single layer in lower temperatures can lead to higher PEC activity compared to that deposited in higher ones. As a result of our PEC characterizations, it is proved that photoconversion efficiency of bare TiO_2_ NWs can be improved by a factor of 1.5 upon coating it with a single ZnO layer at 80 °C. Moreover, considering the fact that this layer is a passivating coating rather than a continuous layer, it also keeps the PEC stability of the design while this feature cannot be obtained in a thick shell layer case. This paper proposes a bottom up approach to control the electron transfer dynamics in a heterojunction design and it can be applied to other metal oxide combinations.

## Introduction

Since the first demonstration of photoelectrochemical (PEC) water splitting by Fujishima and Honda in 1972^1^, a vast variety of materials and design architectures have been exploited over the last five decades to meet the rising demand for larger photoelectrochemical energy conversion^2–6^. However, despite all of these efforts, PEC water splitting remains in its early stages with stable efficiencies far less than 10%, which is the required limit for commercial applications^7^. Among all of the employed semiconductors, titanium dioxide (TiO_2_) has been the core building block of many water splitting cells mainly owing to its high chemical stability, low-cost, non-toxicity, and high resistance to photocorrosion^8–10^. However, the main bottleneck with this metal oxide is its large optical band gap (~3 eV for rutile phase) that makes it inactive under visible and near infrared light irradiation. Moreover, due to the low mobility of carriers in TiO_2_, the collection efficiency of carriers is significantly hampered by surface and bulk defect state mediated recombination. These deficiencies restrict the maximum solar-to-hydrogen efficiency of TiO_2_ to a theoretical value of 2.2%^11^. Therefore, the main field of investigation for TiO_2_ based water splitting cells was to improve the optical and electrical properties of the layer. The ultimate goal in early efforts can be categorized in two main perspectives: 1) extension of light absorption toward longer wavelengths (visible and infrared) to generate higher density of electron-hole pairs, and 2) minimizing the recombination losses to increase the collection efficiency of the carriers.

From optical perspective, elemental doping using metal and non-metal particles^12–24^, hydrogen treatment^25–30^, semiconductor sensitization^31–37^, dye sensitization^38–45^, and plasmonic metal incorporation^46–54^ are examples of the ideas on the development of PEC performance of the TiO_2_. As discussed above, the ultimate goal of these studies is further improvement in light absorption toward longer wavelengths as visible (Vis) and near infrared (NIR) regimes. However, this is not the only requirement that should be satisfied to achieve better PEC performance. In the other words, besides the generation of high density of carriers, we need to collect them through the external circuit to be able to make electricity. Thus, the electrical properties of TiO_2_ should also be developed to increase collection efficiency of the device performance.

Reviewing the PEC water splitting, it can be realized that, for water oxidation process, the photo-induced holes should transfer toward the semiconductor/electrolyte interface before they recombine with their conjugates (which are electrons). Therefore, it is necessary to spatially isolate electron and hole pairs to prolong their lifetimes. This could be achieved by coating a TiO_2_ nanostructure with another metal oxide, which has a proper conduction and valance band position^55–68^. Although, this heterostructure design can selectively isolate electron and hole carriers, the existence of a relatively thick shell layer (with a thickness in the order of tens of nanometers) will increase the path length of holes to reach the semiconductor/electrolyte interface. Considering the short diffusion length of holes (compared to that of electrons), this could significantly block this passage. Moreover, in the case that the shell layer has an amorphous or polycrystalline nature, the existence of bulk and surface trap states can cause further reduction.

Based on a recent theoretical study^69^, the electron transfer rate from a semiconductor coated with a large band gap metal oxide to a representative molecular receptor exponentially declines as the shell layer thickness exceeds 2 Å. It was found that the first atomic layer deposition (ALD) cycle can significantly passivate surface traps without hampering the tunnelling probability of the carrier. However, subsequent cycles have a minimal effect on the passivation of surface traps but engender an exponential decay in the tunnelling probability of the carrier. Therefore, based on the dynamics of photoexcited carriers, the optimum operation can be obtained in a single ALD cycle coated design. Moreover, it was proved that increasing the height of the barrier, which is the energy difference between the photogenerated electron and the bottom of the conduction band of the barrier shell material, results in steeper exponential drops in the tunnelling rate. Later, these findings were experimentally demonstrated in solar cell applications where a sub-nanometer shell layer could significantly boost the device efficiency^70–73^. In a recent study, we showed that a single ALD cycle of ZnO can significantly improve the photocatalytic behavior of the TiO_2_ NWs over the degradation of dye as an organic pollutant^74^. It was found that a single ALD cycle can efficiently passivate the surface traps while keeping the valence band holes’ contribution up via tunneling across the ultrathin ZnO layer. A substantial improvement in electron transfer dynamics can be attained by tuning the band alignment between the core and shell layers. Reducing the barrier height for hole tunnelling can be another factor which could further increase the carrier injection probability. This could be achieved by adopting proper deposition temperature for ALD ZnO layer. Thus, it is envisioned that deposition of a single ALD cycle of ZnO in a proper temperature could significantly increase the photocatalytic performance of the design.

In this work, we experimentally demonstrate that a single ALD cycle coating of ZnO shell can improve the PEC water splitting performance of hydrothermally grown TiO_2_ NWs core. It was found that such an angstrom thick layer can efficiently passivate trap states in the surface of TiO_2_ NWs. To further optimize the ZnO coating, the deposition temperatures have been chosen as 80 °C, and 250 °C. It was found that 80 °C TiO_2_-ZnO has the highest PEC activity compared to those of 250 °C TiO_2_-ZnO and bare TiO_2_ samples. This was attributed to smaller energy difference between valance bands of TiO_2_ and ZnO which this in turn increases the tunnelling probability of photogenerated holes. The characterization results also prove these statements that this single ALD cycle of ZnO has efficiently passivated surface traps of TiO_2_ NW. Moreover, by altering the deposition temperature, the band alignment between TiO_2_ and ZnO layers can be tuned in a controlled manner to facilitate the carriers transfer dynamics. Finally, due to the ultrathin nature of ZnO coating, the proposed structure has retained its chemical stability almost intact over a long duration of light irradiation.

## Results and Discussion

**Figure 1:**
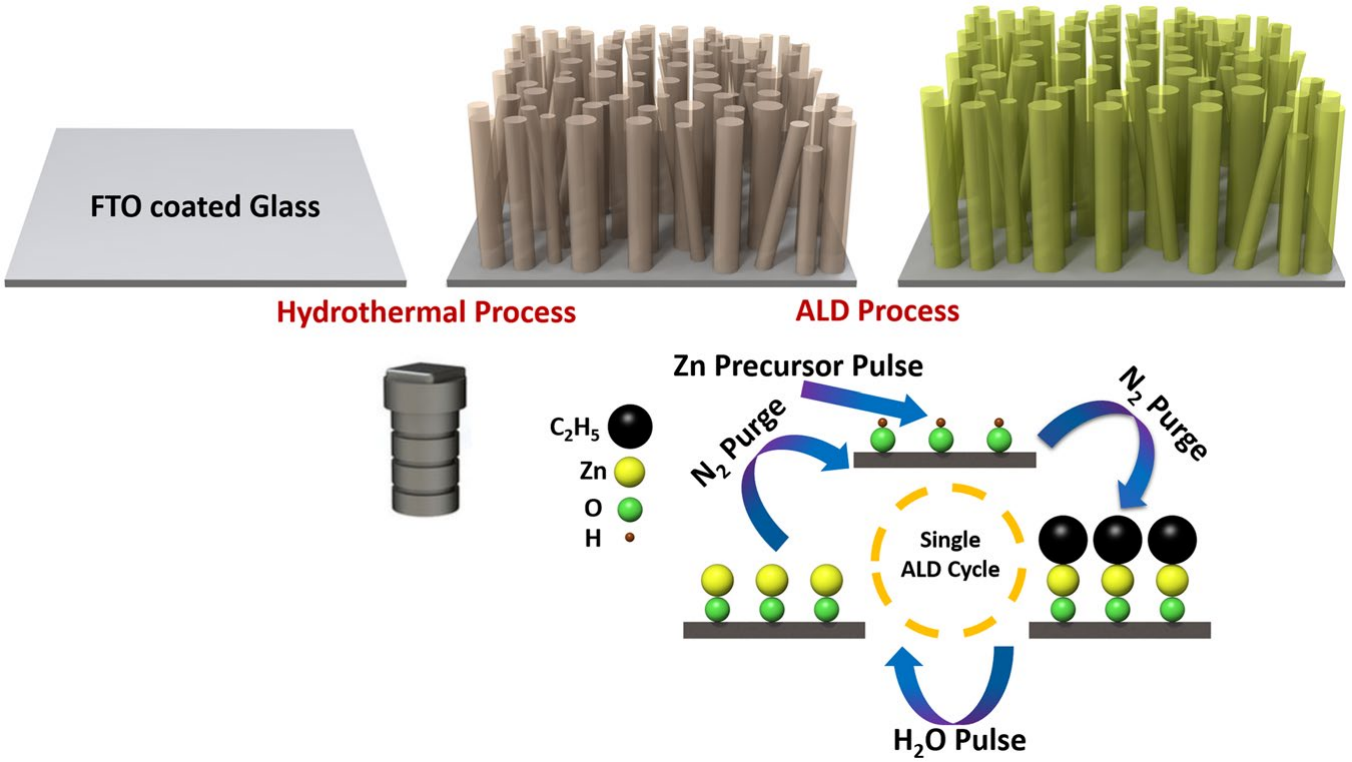
Preparation route of bare and ZnO coated TiO_2_ NWs. Hydrothermal process for a duration of 4 hours at 180 °C is utilized to grow TiO_2_ NWs and ALD method is conducted to coat the NWs with single ZnO layer at two different temperatures of 80 °C and 250 °C.

**Figure 2:**
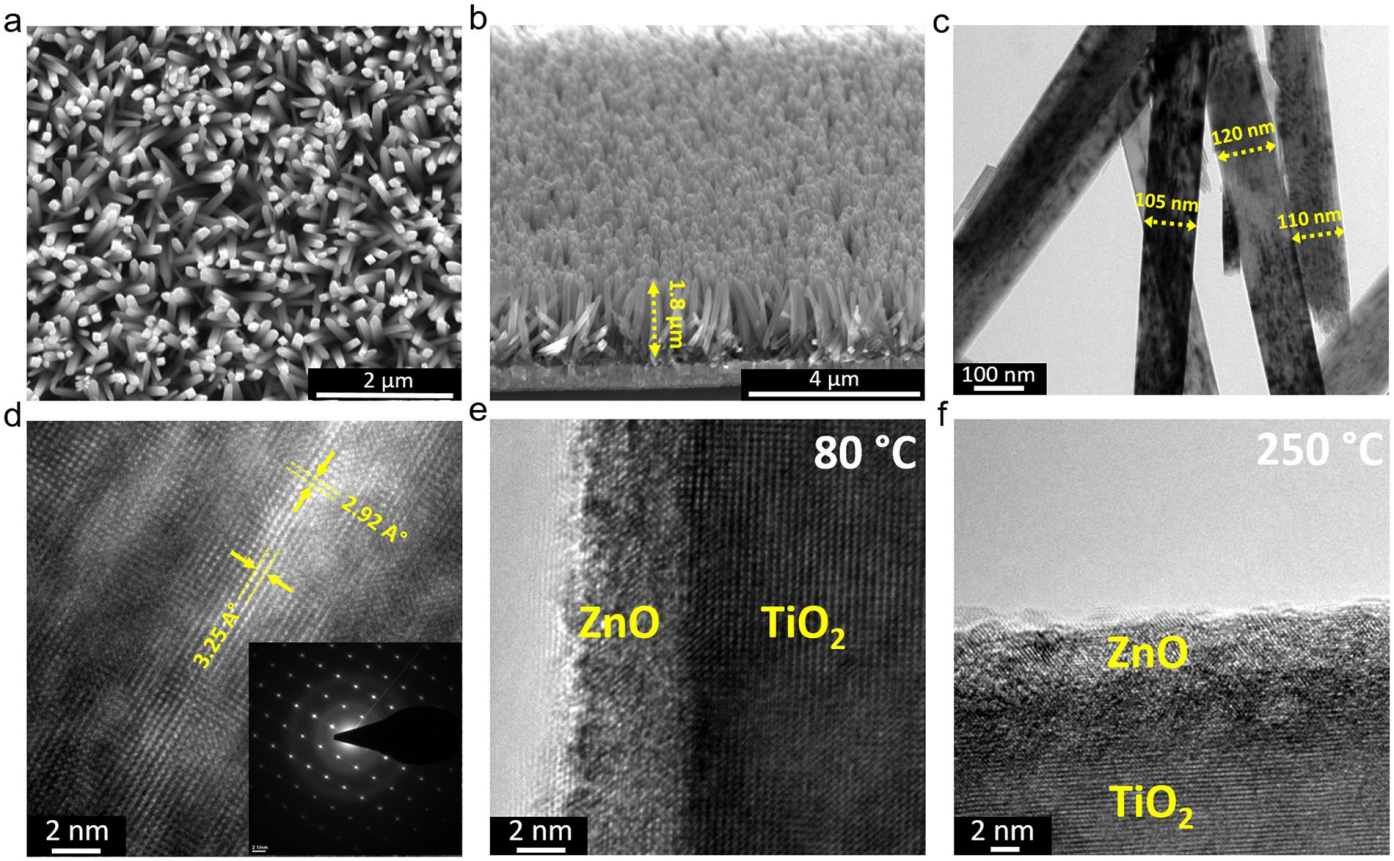
The (**a**) top and (**b**) cross sectional SEM images of the prepared TiO_2_ NWs which shows the formation of tightly packed TiO_2_ NWs with a length of 1.8 µm. (**c**) The TEM images of the NWs proving the diameter of NWs to be about 110 nm and (**d**) HRTEM image showing formation of single crystalline TiO_2_ NWs. The inset shows the SAED pattern of the sample. HRTEM images of (**e**) 80 °C, and (**f**) 250 °C 50 cycle ZnO coated TiO_2_ NWs.

Figure 1 schematically represents the fabrication route of the proposed TiO_2_-ZnO structure. The details of materials, photoanode preparation, and characterizations have been provided in supplementary information. In summary, similar to our previous works^75,76^, we first synthesize TiO_2_ NWs using hydrothermal method at 180 °C for a duration of 4 hours. Later, these NWs were annealed at 450 °C for 2 hours to improve their mobility and to remove the possible surfactants from their surface. Finally, the samples were transferred into an ALD chamber to coat them with single ALD cycle of ZnO shell at two different temperatures of 80 °C and 250 °C. These temperature values are lower and upper limits of our ALD system. Below the lower limit, the coating could be incomplete due to slow reaction kinetics and above the upper limit cannot be provided with our ALD system. The details of the ALD process are provided in the experimental section. Figure 2a depicts the top SEM image of the synthesized ZnO coated TiO_2_ NWs. As this graph clearly implies, the obtained NWs have a high packing density and random orientation that enables them to efficiently harvest the light. Moreover, the cross sectional SEM image of the architecture reveals that the NWs have an approximate length of 1.8 µm (see Fig. 2b). As depicted in Fig. 2c, TEM image shows that the mean diameter of the NWs is about 110 nm. Moreover, the crystallinity phase of the material can be scrutinized by looking at its high resolution TEM (HRTEM) image. HRTEM image, shown in Fig. 2d, demonstrates the formation of single crystalline TiO_2_ NWs with inter-planar distances of d_110_ = 3.25 Å and d_001_ = 2.92 Å that is consistent with those of rutile phase of TiO_2_. This can be further confirmed by the selected area electron diffraction (SAED) pattern of a NW examined along the [110] zone axis, as shown in the inset of this panel. It should be mentioned that due to the ultrathin thickness of single ALD cycle of ZnO (~1 Å), it is not possible to discern such coating in the HRTEM image. However, a better understanding on the morphology of the ZnO shell can be acquired by coating the TiO_2_ NWs with a thicker ZnO shell layer. For this aim, the ZnO deposition is carried out for 50 ALD cycles at two different temperatures of 80 °C and 250 °C in order to obtain ZnO bulk structure and in the graphs the abbreviation (80 °C ZnO and 250 °C ZnO) will be used. According to the HRTEM images, Fig. 2e–f, a uniform coating has been achieved on NWs’ surface that is due to the nature of ALD process. A closer look can provide us important information on the crystallinity phase of the synthesized ZnO shells. As shown in these panels, the sample that has been prepared at the deposition temperature of 250 °C have better crystallinity compared to that of 80 °C. More specifically, a higher deposition temperature could facilitate the formation of larger grains where more atoms are aligned in a specific direction. These grains are smaller for the case of a lower deposition temperature. Therefore, it can be envisioned that in the case of single ALD cycle deposition, bigger ZnO crystalline particles are wrapped around the TiO_2_ NWs. Consequently, this offers a facial bottom up approach to tune the ZnO crystalline size and this in turn could tune the electronic band structure and the optical properties of the material.

**Figure 3:**
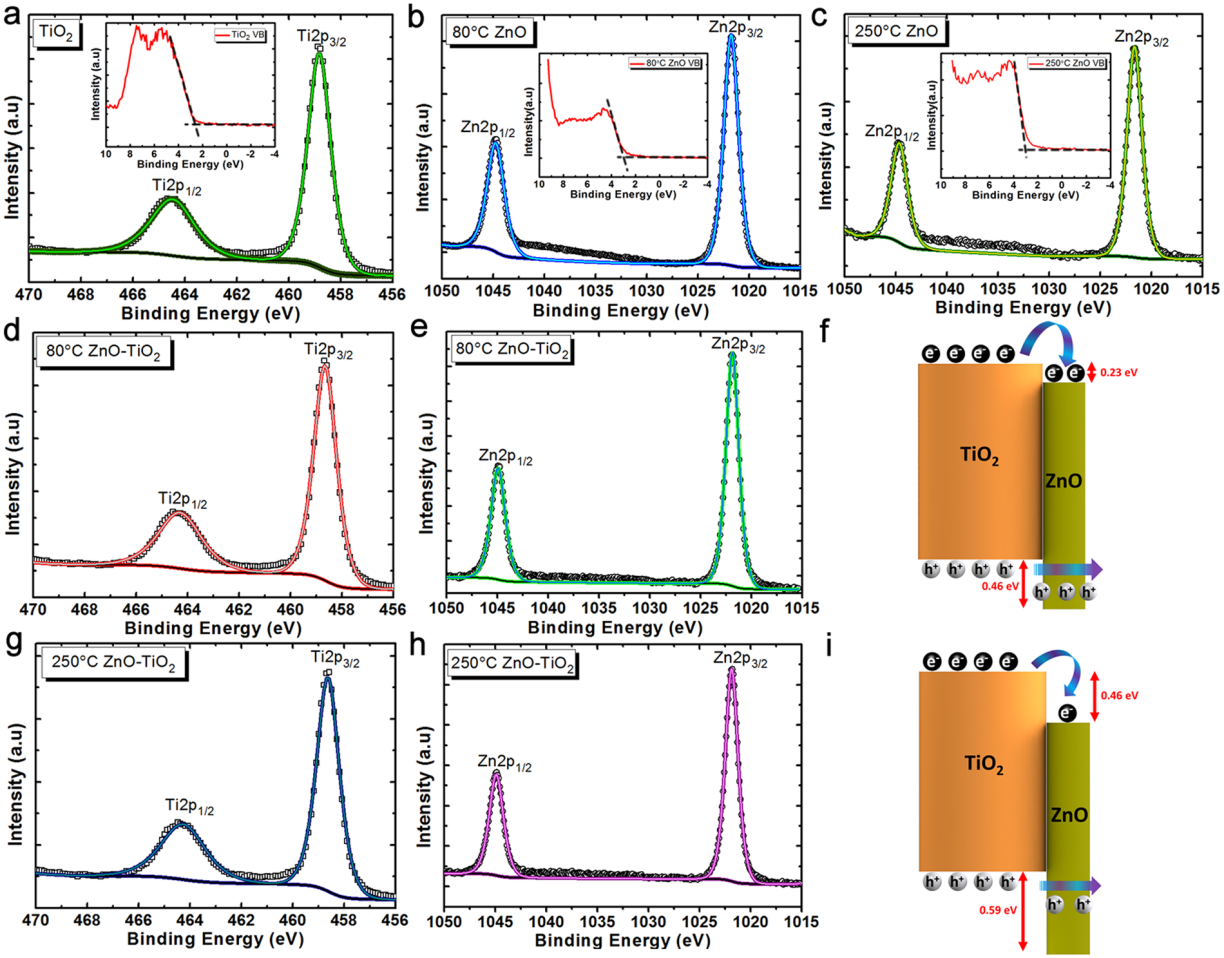
(**a**) Ti2p spectra of the bare TiO_2_ NWs, Zn2p spectra of (**b**) 80 °C deposited ZnO bulk, and (**c**) 250 °C deposited ZnO bulk. The inset shows the energetic positions of VBMs for these three samples. The (**d**) Ti2p, and (**e**) Zn2p spectra of 80 °C TiO_2_-ZnO sample and (**f**) corresponding band alignment of 80 °C TiO_2_-ZnO. The (**g**) Ti2p, and (**h**) Zn2p spectra of 250 °C TiO_2_-ZnO sample and (**i**) corresponding band alignment of 250 °C TiO_2_-ZnO.

To confirm this statement, X-ray photoelectron spectroscopy (XPS; Thermo, K-Alpha monochromated high-performance XPS spectrometer) measurements are carried out to monitor the composition of each layer and their interface. The elemental composition data have been depicted in Table S1. For this aim, we first carried out the XPS measurement on bare TiO_2_ NWs, and bulk ZnO deposited at 80 °C and 250 °C as plotted in Fig. 3a–c. From bare TiO_2_ NWs spectrum, the core level binding energy of Ti2p_3/2_ is located at 458.82 eV. Moreover, the binding energies of Zn2p_3/2_ for 80 °C and 250 °C deposited bulk ZnO samples are placed at 1021.74 eV, and 1021.64 eV, respectively. In addition, we need to determine the energetic positions of valance band maxima (VBM) for each sample. These values are calculated by intersection of the linear portion with the background line of each spectrum. The VBM values are found to be 2.71 eV, 2.88 eV, and 2.92 eV for bare TiO_2_ NWs, 80 °C and 250 °C bulk ZnO, respectively. Later, the XPS measurements are conducted on single cycle coated 80 °C TiO_2_-ZnO and 250 °C TiO_2_-ZnO heterostructure to examine the core level spectra of these samples. As plotted in Fig. 3d–e, the energetic position of Ti2p_3/2_ and Zn2p_3/2_ are placed at binding energies of 458.65 eV and 1021.86 eV for the 80 °C TiO_2_-ZnO sample. These values are found to be 458.63 eV and 1021.83 eV for 250 °C TiO_2_-ZnO as exhibited in Fig. 3g–h. Besides these values, the optical band gap (*E*_*g*_) for each layer is needed to find the band alignment between these two semiconductors. To obtain the band gaps, we have first adopted a combinational spectroscopy method on each sample to determine the absorption profiles at a wavelength range of 320 nm to 500 nm. For this aim, we first obtained the transmission spectra (T) of the samples using UV-Vis-NIR spectroscopy. It should be mentioned that NWs with dimensions comparable with incident light wavelength can significantly scatter the light and block its transmission. Therefore, the spectroscopy results from such samples may not have been accurate. To avoid this issue, we synthesized shorter TiO_2_ NWs in which the hydrothermal process is conducted for duration of 1 hour. While 4 hour grown NWs have a quite hazy appearance (that shows their high level of light scattering), these shorter samples were transparent. Later, the reflection (R) of the design is measured in the required range, using an ellipsometry device. To suppress the effect of reflection from the back surface, translucent (cloudy looking) Scotch tape is adhered on the bottom of the samples. Finally, the absorption (α) spectra of these samples are obtained using the following formula of α = 1 − R − T. Figure S1a plots the absorption spectra for each of these layers. From this data, the Tauc plot is calculated using the formula of (αω)^0.5^ where α is the absorption coefficient and ω is the angular frequency and depicted in Fig. S1b. The optical band gap of bare TiO_2_, 80 °C and 250 °C deposited ZnO bulk is estimated by the intersection of the linear part of the graph with the x-axis. As the output of this characterization, the optical band gaps of TiO_2_, 80 °C ZnO, and 250 °C ZnO were found to be 2.88 eV, 3.11 eV, and 3.01 eV. Having all of these values, we can determine the band alignment between TiO_2_ and ZnO by using the following formula^72^:

1 $${\rm{\Delta }}{E}_{CL}={({E}_{ZnO}-{E}_{Ti{O}_{2}})}_{{{\rm{TiO}}}_{2}/{\rm{ZnO}}{\rm{HJ}}}$$

2 $${\rm{\Delta }}{E}_{V}(ZnO/Ti{O}_{2})={({E}_{CL}-{E}_{VBM})}_{\tfrac{bulk}{Ti{{\rm{O}}}_{2}}}-{({E}_{CL}-{E}_{VBM})}_{\frac{bulk}{ZnO}}+{\rm{\Delta }}{E}_{CL}$$

3 $${\rm{\Delta }}{E}_{C}(ZnO/Ti{O}_{2})={({E}_{{g}_{Ti{O}_{2}}}-{E}_{{g}_{ZnO}}+{\rm{\Delta }}{E}_{V})}_{Ti{O}_{2}/{\rm{ZnO}}{\rm{HJ}}}$$

where Δ*E*_*CL*_ is the energy difference between the Zn2p (*E*_*ZnO*_) and Ti2p (${E}_{Ti{O}_{2}}$) core levels (CLs) in the TiO_2_–ZnO (heterojunction) NW sample, ${({E}_{CL}-{E}_{VBM})}_{\tfrac{bulk}{Ti{{\rm{O}}}_{2}}}$is the binding energy difference between the CL and the valance band maximum (VBM) for bare rutile TiO_2_ NWs, ${({E}_{CL}-{E}_{VBM})}_{\tfrac{bulk}{ZnO}}$is the binding energy difference between the CL and the valance band maximum (VBM) for bulk thick bare ZnO coating, ${E}_{{g}_{Ti{O}_{2}}}$ is the optical band gap of the TiO_2_ NWs,${E}_{{g}_{ZnO}}$ is the optical band gap of the bulk ZnO layer, Δ*E*_*C*_ and Δ*E*_*V*_ are the energy difference between conduction and valance bands of TiO_2_ and ZnO in the heterojunction design. Δ*E*_*V*_ is found to be 0.46 eV for 80 °C TiO_2_-ZnO and 0.59 eV for the case of 250 °C. Moreover, the Δ*E*_*C*_ values are 0.23 eV and 0.46 eV for these cases. Thus, these results prove that the coating of a ZnO layer at lower temperatures would shift the energetic positions of the bands toward positive values. This phenomenon can be explained taking the TEM results into consideration. In the previous part, we showed that the size of crystalline regions is smaller for the case of lower deposition temperature. Consequently, due to the quantum confinement effects, smaller crystalline particles can provide a positive shift in the energetic positions of both conduction and valance bands^77^. Therefore, these XPS results are in line with our expectations from the TEM measurement. Based on previous studies calculations^69^, it was demonstrated that the increase in the height of the barrier, which is the energy difference between the photogenerated hole and the top of the valence band of the shell material in our case, results in steeper exponential drops in the tunneling rate of carriers. From this perspective, the 80 °C deposited sample has a better carrier transfer dynamics and a higher PEC water splitting activity can be expected from this sample compared to that of the 250 °C one.

**Figure 4:**
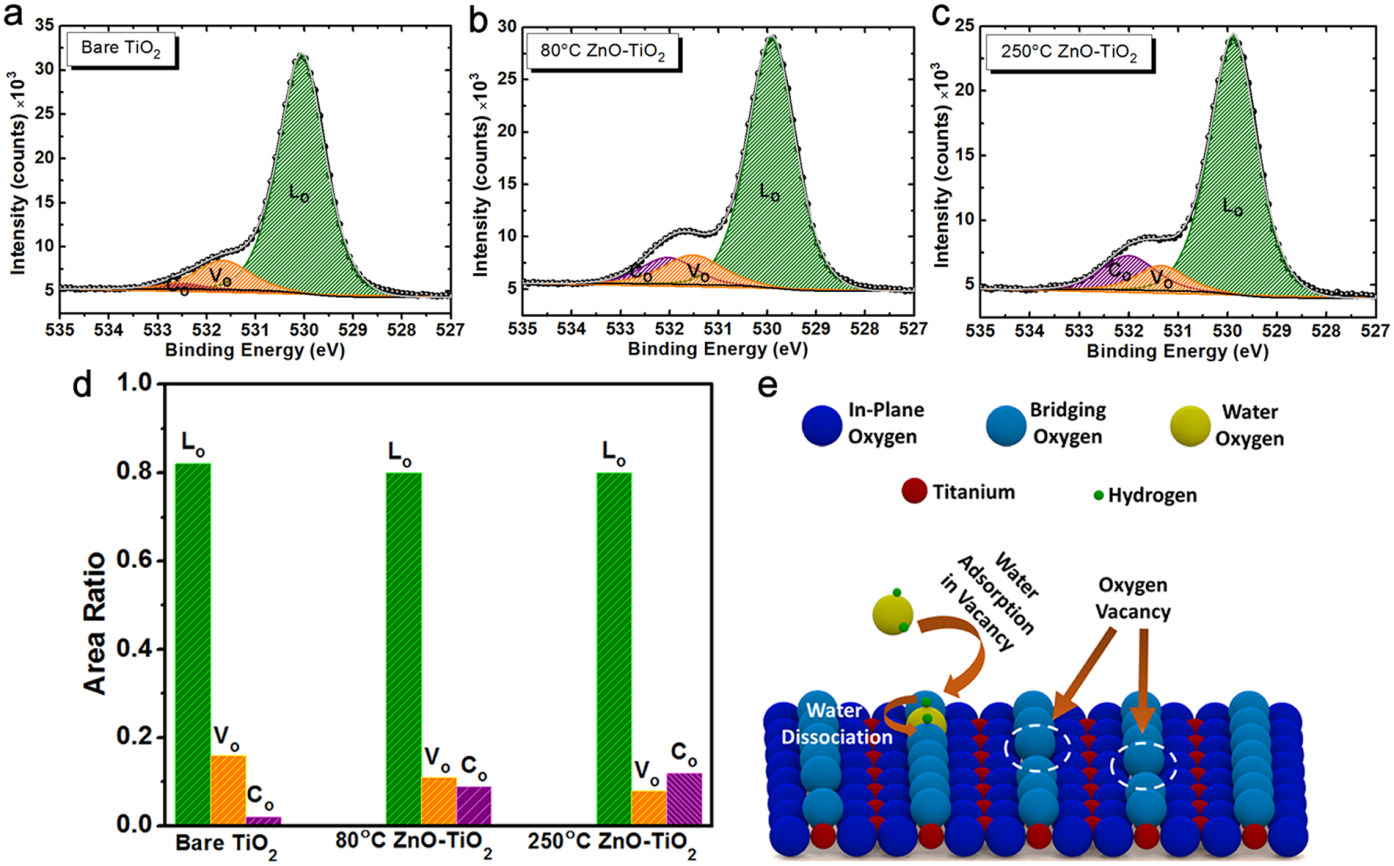
(**a**) The XPS O1s spectra of (**a**) bare TiO_2_, (**b**) 80 °C TiO_2_-ZnO, and (**c**) 250 °C TiO_2_-ZnO that have been deconvoluted into three Gaussian peaks attributed to lattice oxygen (L_O_), oxygen vacancy (V_O_), and chemisorbed oxygen (C_O_). (**d**) The calculated area ratios of L_O_, V_O_, and C_O_ for all three samples. (**e**) The proposed mechanism for the passivation of surface traps upon coating with ZnO layer during ALD process.

The XPS measurement is not only used to provide information about the electronic band structure of the design but also it can be employed to study the surface properties of the layer. For this aim, the existence of surface defects, the O1s spectra of the samples can be analyzed and compared; hence, the core-level O1s spectrum of the bare and ZnO coated TiO_2_ NWs sample can be deconvoluted into three Gaussian peaks, as explained in our previous studies^72,74^. The major peak centered at 530 eV is attributed to the lattice oxygen (L_O_) bonded to Ti^+4^ ions. The other Gaussian components with center binding energies of 531.4 eV and 532 eV are assigned to oxygen vacancy/defects (V_O_) and chemisorbed/dissociated oxygen species (C_O_) on the surface of NW. Figure 4a–c plots the O1s spectra for three different cases of bare TiO_2_, 80 °C TiO_2_-ZnO, and 250 °C TiO_2_-ZnO, respectively. As this graph clearly implies, in the case of bare TiO_2_ NW, the portion related to chemisorbed oxygen is negligible and relatively large density of the oxygen vacancy defects are probed on the surface. However, upon the coating of these NWs with a single ALD cycle of ZnO, the peak attributed to V_O_ gets smaller and C_O_ related peak gets larger amplitude. A more suitable qualitative comparison can be acquired by defining the ratio of areas related to each component. Figure 4b plots these ratios for all three samples. Based on this panel, the deposition of ZnO could reduce the V_O_ related portion from 0.16 for bare TiO_2_ to 0.11, and 0.09 for 80 °C, and 250 °C TiO_2_-ZnO samples, respectively. These values for C_O_ cases are 0.02, 0.09, and 0.12, respectively. Therefore, from these findings, it can be concluded that the deposition of ZnO has passivated a part of vacancy defects. This phenomenon can be addressed by looking at the details of ALD process. In the ALD method, TiO_2_ NWs are first exposed into water vapor as the source of oxygen. Based on the calculation of free energy by classical nucleation theory, the oxygen derived radicals have the tendency to be chemisorbed at the imperfections such as oxygen vacancies^72,74^. Thus, it is expected that water molecules are attached into oxygen vacant positions and water-induced H ion is subsequently transferred into neighboring bridging oxygen atoms. This process has been schematically shown in Fig. 4e. This explanation is also in accordance with previous theoretical estimations that the first ALD cycle can significantly passivate surface traps while successive cycles have only a weak contribution^69,71–74^. This statement has been further proven by exposing titania NWs only to water pulse. The O1s spectra for TiO_2_ NWs exposed only by single water ALD cycle at 250 °C is shown in Fig. S2. Comparing with the O1s spectrum of bare TiO_2_ NWs, we can clearly deduce that exposing NWs to water pulse has passivated the surface traps. However, the peak attributed to chemisorbed oxygen have been intensified. This is in agreement with our explanations in Fig. 4e.

**Figure 5:**
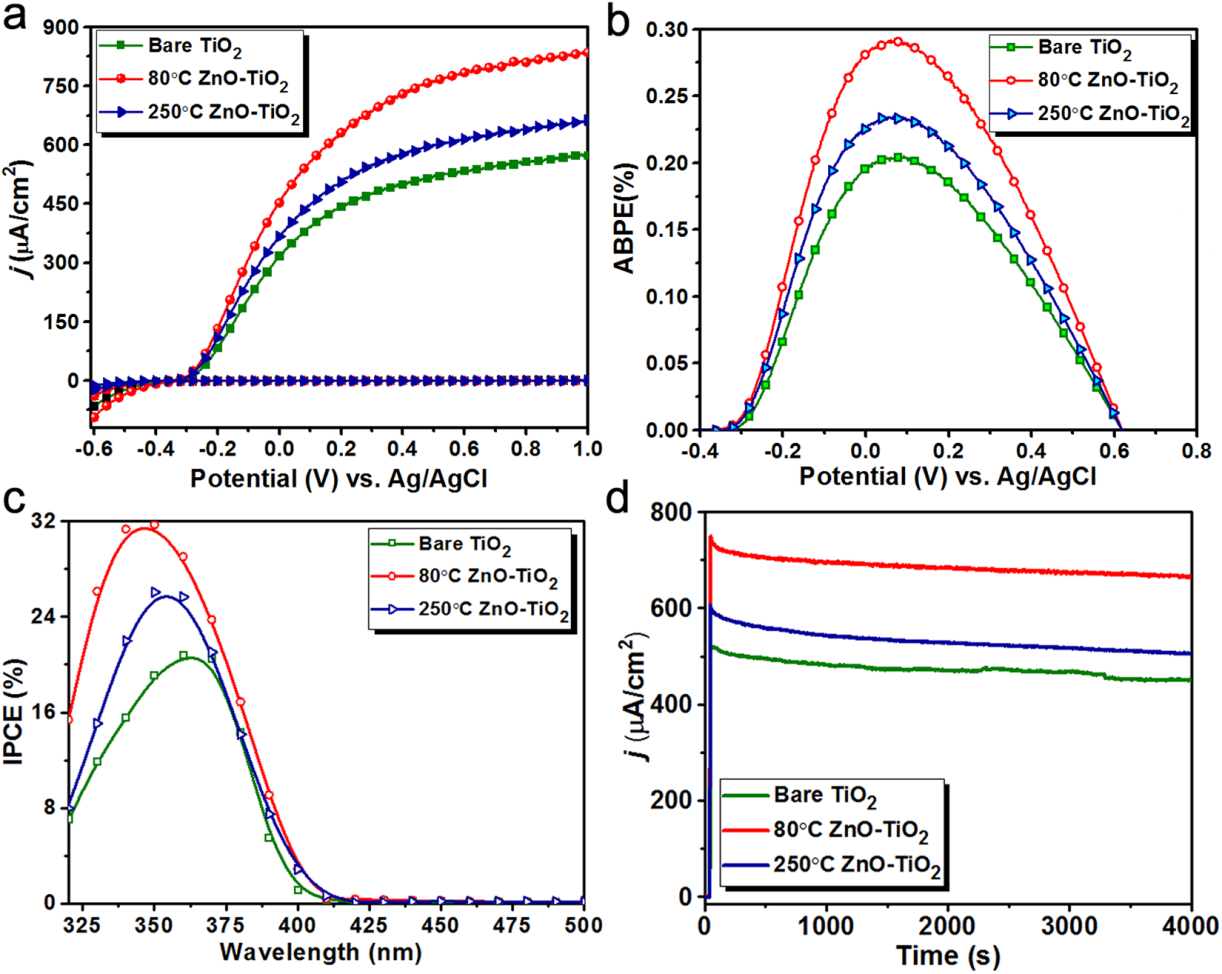
The bare TiO_2_, 80 °C TiO_2_-ZnO, and 250 °C TiO_2_-ZnO samples (**a**) *j-V* plots measured under dark and light, (**b**) their corresponding ABPE values. (**c**) The IPCE values in a wavelength range of 320 nm to 500 nm. (**d**) *j-t* plots under solar irradiation for 4000 seconds at 0 V (vs Ag/AgCl). Measurement conditions are in N_2_-saturated 0.5 M PBS (pH 7.02) for fabricated samples.

Based on the above-mentioned characterization results, it can be envisioned that the application of a single ALD ZnO layer could substantiate the PEC performance of the TiO_2_ NWs. To evaluate the PEC performance of the samples, linear sweep voltammetry (LSV) measurements are carried out on the samples in both dark and under illumination conditions. For this aim, the photoanodes are placed inside PBS aqueous electrolyte (pH 7.02) in a three-electrode cell. At an applied potential of 0.62 V vs Ag/AgCl (corresponded to 1.23 V vs RHE), the photocurrent densities of the bare, 80 °C TiO_2_-ZnO, and 250 °C TiO_2_-ZnO samples are recorded to be 534 µA/ cm^2^, 785 µA/cm^2^, and 618 µA/cm^2^, respectively. Therefore, in line with our expectations, the single ALD cycle ZnO treatment has significantly improved the activity of bare TiO_2_ NWs AND 80 °C TiO_2_-ZnO photoanode sample has the highest activity. To have a better qualitative comparison, the applied bias photon-to-current efficiency (ABPE) is calculated for all these three samples using the following formula:

4 $$ABPE( \% )={[\frac{|{J}_{ph}|\times ({V}_{th}-|{V}_{b}|)}{{P}_{total}}]}_{AM1.5}\times 100\,$$

where *J* is the photocurrent density at the specified applied potential (mA/cm^2^), *V*_***th***_ is the theoretical water-electrolysis voltage (1.23 V), *V*_*bias*_ is the applied potential (V vs RHE), and P is incident power density of the light (mW/cm^2^). According to the Fig. 5b, the maximum values for ABPE (recorded at applied bias value of 0.06 V vs Ag/AgCl) are 0.20%, 0.23%, and 0.29% for bare TiO_2_, 80 °C, and 250 °C TiO_2_-ZnO samples, respectively. Therefore, these results prove that deposition of a single ALD cycle at the proper temperature can improve the photoconversion efficiency of the bare TiO_2_ NW sample with a factor of approx. 1.5. This proves the significance of the semiconductor/electrolyte interface in PEC WS cell, where the efficient passivation of surface defects could promote the water splitting process. Moreover, the smaller energy barrier in the case of an 80 °C deposited sample will increase the tunneling probability of the generated holes. This could be further confirmed by looking at incident photon conversion efficiency (IPCE) values for the proposed samples. Figure 5c shows the IPCE curves for the bare and ZnO coated samples. As expected, all three samples have only their response in the ultraviolet (UV) range which is in line with the fact that TiO_2_ has no absorption in the visible regime. However, single ALD ZnO coated samples have larger IPCE values compared to that of bare TiO_2_ NWs. Considering the fact that the deposition of an ultrathin ZnO shell cannot significantly change the absorption capacity of the TiO_2_ NWs, this enhancement is attributed to improvement in charge collection and transport capability of the treated samples. Another important factor that defines the feasibility of a photoanode for the long-term applications is its photocurrent stability. To evaluate this parameter, samples have been exposed to incident solar spectrum for 4000 seconds and the decay of the photocurrent densities have been recorded throughout this time. As clearly plotted by Fig. 5d, all three samples have relatively stable response under light illumination. The small reduction in the photocurrent values is due to the formation of bubbles in the surface of photoanode. This is an important feature that cannot be realized for thicker ZnO layers where a significant reduction in the photocurrent values is recorded due to the photocorrosion of the ZnO layer^59,60^. However, in our case, the ZnO is rather a passivating coating that terminates surface defects rather than being a continuous layer. Therefore, while it improves the PEC activity of the TiO_2_ NWs, it does not diminish the chemical stability of the system. Therefore, all the obtained characterization findings prove the raising of the PEC performance of TiO_2_ NWs upon coating it with single ALD ZnO layer. However, further characterizations are required to elucidate the differences between 80 °C and 250 °C treated TiO_2_-ZnO samples.

**Figure 6:**
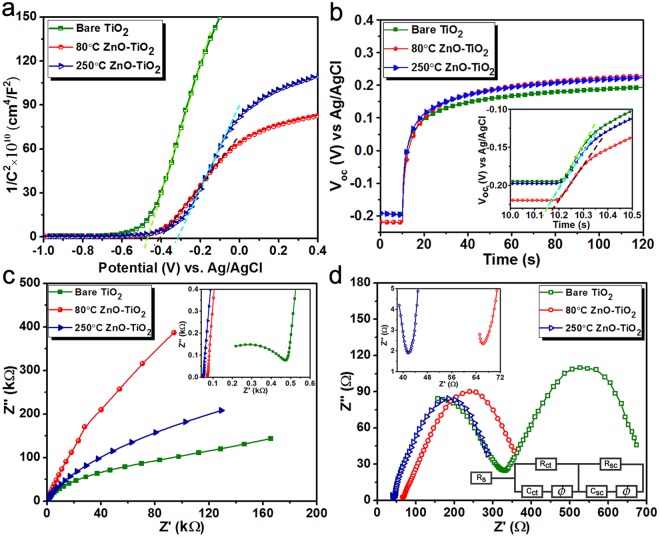
The bare TiO_2_, 80 °C TiO_2_-ZnO, and 250 °C TiO_2_-ZnO samples (**a**) The Mott-Schottky profiles, (**b**) The OCPD measurements with the inset shows the magnified image of the measurement at the exact moment of light cut off, (**c**) The EIS spectra in dark and (**d**) The EIS spectra under light irradiation conditions. The (**c**,**d**) insets exhibit a magnified image of these spectra. Moreover, the circuit model used for data fitting is also presented in the (**d**) inset.

One of the useful characterizations that can provide detailed information about the interface of a photoanode is Mott-Schottky measurement. By performing electrochemical impedance spectroscopy (EIS) measurements in a three-electrode cell with varying the applied voltage bias, the Mott-Schottky profiles have been calculated and depicted in Fig. 6a. The measurement has been conducted at a high frequency of 5 KHz to ensure that the contribution of surface state capacitance can be safely neglected. It is known that this profile can be fitted into following relation which is called Mott-Schottky equation:

5 $$\frac{1}{{{C}_{SC}}^{2}}=\frac{2}{({{\rm{\varepsilon }}}_{{r}}{{\rm{\varepsilon }}}_{o}{A}^{2}e{N}_{d})}\times ({V}_{Applied}-{V}_{fb}-\frac{{k}_{B}T}{e})\,$$

where CSC is the space charge layer capacitance, *e* is the charge of an electron, *ε* is the relative permittivity of the semiconductor which TiO_2_ in our case, *ε*_0_ is the vacuum permittivity, *A* is the surface area, *N*_*d*_ is the majority carrier density, *V*_*Applied*_ is the applied voltage (vs. Ag/AgCl), *V*_*fb*_ is the flat band potential, *k*_*B*_ is Boltzmann constant, and *T* is the absolute temperature. *V*_*fb*_ can be found by the interpolation of the linear fitting of the transition region of Mott-Schottky plot with the x-axis. Based on the profiles obtained in Fig. 6a, the *V*_*fb*_s are extracted as −0.48 V, −0.43 V, and −0.35 V (vs Ag/AgCl) for bare, 80 °C TiO_2_-ZnO, and 250 °C TiO_2_-ZnO, respectively. Therefore, the introduction of a single ZnO layer into TiO_2_ NWs imposes a positive shift in the energetic location of *V*_*fb*_. This shift is more pronounced for the case of 250 °C TiO_2_-ZnO. Essentially, the flat band potential is the potential in which the surface band bending is disappeared and this is equivalent to the Fermi level in semiconductors. On the other hand, considering the fact that the Fermi level of an n-type semiconductor is located within the band gap, slightly below its conduction band edge, it can be expected that this value gives a fair measure of the position of conduction band minima for three different cases. From the XPS results, we found the band alignment in the TiO_2_-ZnO interface for two different cases of 80 °C and 250 °C deposited ZnO samples. It is worth noting that the energetic location of the sample treated at 80 °C is placed at more positive energy levels compared to that of 250 °C. Therefore, the obtained Mott-Schottky results confirm those of the XPS ones in which a lower deposition temperature could offer a more suitable band alignment for the TiO_2_-ZnO NW design. Moreover, the majority carrier density (*N*_*d*_) of the semiconductor photoanode can be extracted from the slope of this graph. A smaller slope proves the existence of the high density of free carriers and higher electrical conductivity which this, in turn, means a much higher collection efficiency of photo-induced electrons. It should be mentioned that an absolute value of carrier densities can only be attained in the case of a flat planar photoanode in which the geometrical area is equal to the active surface area. However, in our case which is made of a tightly packed TiO_2_ NWs, the real surface area of the electrode is much higher than that of the planar geometrical area. However, this slope can still be used to compare the carriers’ densities in the proposed three different photoanodes. Taking the Mott-Schottky profile of bare TiO_2_ NW as the normalization factor, the slopes for 80 °C and 250 °C TiO_2_-ZnO heterostructure are found to be 0.36 and 0.59 times of that of bare one. This means that the majority carrier density of 80 °C TiO_2_-ZnO photoanode is 2.77 times larger than that of bare while this enhancement factor is 1.69 for the case of 250 °C TiO_2_-ZnO. This is mostly attributed to the passivation of surface defects of TiO_2_ NWs upon coating the single ALD cycle of ZnO in which this passivation could mitigate carriers’ recombination and this consequently results in higher electrical conductivity. In addition, the more energetically suitable band alignment in the case 80 °C treated sample can provide more efficient electron-hole pair separation in the TiO_2_-ZnO interface and accordingly higher density of free carriers.

Further insight into the interfacial charge transfer in TiO_2_-ZnO photoanodes can be acquired by means of open-circuit potential decay (OCPD) measurement. For this aim, the photoanode sample is exposed to solar spectrum irradiation to stabilize the open-circuit potential (V_OC_). Afterward, the illumination is turned off and the subsequent decay of photovoltage is recorded as a function of the time. As illustrated in Fig. 6b, all the photoanodes experience a negative shift under light illumination compared to that in dark condition. This is due to the fact that upon the excitation of a semiconductor, photo induced electrons are transferred to conduction band and consequently Fermi level rapidly shifts to more cathodic potentials. Once illumination is terminated, the *V*_*OC*_ returns to its initial value in the dark condition via the recombination of photogenerated carriers. The accumulated electrons are recombined through two main paths: 1) band-to-band transition in which the electrons in conduction band recombine with holes in valance band which is the dominant recombination process in the bulk of semiconductor, 2) band-to-surface transition where the recombination is mediated with shallow and deep surface traps and this kinetically faster compared to an earlier one. The decay rate of this process is a measure of the kinetics at the semiconductor/electrolyte interface and the lifetime of the electrons can be calculated by the following equation:

6 $${\tau }_{n}=\frac{-{k}_{B}\times T}{e}{(\frac{d{V}_{OC}}{dt})}^{-1}\,$$

where τ_n_ is electron lifetime, *k*_*B*_ is the Boltzmann constant, *T* is the temperature (room temperature of about 300 K), *e* is the charge of an electron, and *V*_*oc*_ is the open-circuit voltage at a specific time t. As already mentioned, the existence of two recombination mechanism imposes two different exponential decay rates in which the portion related to band-to-surface recombination has faster dynamics with abrupt decay. Therefore, the slope of *V*_*oc*_ decay in the exact moment after the light turns off could give us the right information about the interfacial charge transfer process. The inset of Fig. 6b shows a magnified version of the OCPD profile at the moment of light cut off. As can be clearly seen from this figure, the slope is the smallest for the case of 80 °C TiO_2_-ZnO and it gradually increases for 250 °C TiO_2_-ZnO and bare TiO_2_ photoanodes. Using the above equation, the electron lifetime is calculated to be 0.04 s, 0.052 s, and 0.048 s for bare TiO_2_, 80 °C TiO_2_-ZnO, and 250 °C TiO_2_-ZnO, respectively. These results again confirm the fact that a proper charge separation can be realized in the case of ZnO coated TiO_2_ NWs. This is mainly due to the passivation of surface traps and the existence of a proper band alignment between ZnO and TiO_2_ layers. Moreover, the smaller energy difference between the conduction band minima of ZnO and TiO_2_, in the case of 80 °C deposited ZnO, makes the electron injection more likely compared to that of 250 °C and, therefore, the electron lifetime is further prolonged. It should be mentioned that the existence of adsorbed chemical species (during the preparation of the sample) can also affect potential of photoelectrodes. However, as mentioned in the method section of the paper, the samples were rinsed with DI water to remove residual solvent, and calcined at 450 °C for 2 hours in a tube furnace under air. By this way, all chemical residues are removed from the sample.

A deeper insight into the charge transfer process inside the proposed photoanodes can be obtained by employing an EIS spectroscopy measurement that is carried out in a frequency range of 0.1 Hz-10 KHz using an AC amplitude of 10 mV at the open circuit potential of the system. The Nyquist plots, where x and y-axes are the real part (*Z*′) and the negative of imaginary part (−*Z*″) of the impedance, respectively, represents the charge-transfer process in the photoelectrochemical system. Figure 6c exhibits the Nyquist profiles for these three different photoanodes in the dark condition. Plots have a semicircle shape at high frequencies and an inclined semi straight lines is the profile that is recorded in lower frequency regions. At large frequency limits, the intersection point of Nyquist plot with the x-axis represents the internal resistance of the system (*R*_*s*_), including the intrinsic resistance of the photoelectrode active material, bulk resistance of the electrolyte solution, and contact resistances. Moreover, the diameter of the semicircle depicts the interfacial charge transfer resistance (*R*_*ct*_) at the interface of semiconductor/electrolyte. At the low frequency limits, the semi linear line is inversely corresponded to the Warburg impedance or diffusion resistance. Therefore, a steeper line means lower ion diffusion resistance. As plotted in the inset of Fig. 6c, the Bare TiO_2_ has a much larger semicircle compared to those of ZnO coated samples indicating the lower *R*_*ct*_ values for these treated samples, which is an expected result when taking the previous results into consideration. At the other end of the scale, the straight line slope is the largest for 80 °C TiO_2_-ZnO and it declines gradually for 250 °C TiO_2_-ZnO, and TiO_2_ NWs. The better ion diffusion can be ascribed to the enhanced conductivity possessed in the core-shell structure. Upon shining these samples with solar irradiation, however, the impedance values significantly reduce, which is due to the photogeneration of electron-hole pairs. The Nyquist plot under light irradiation is made of two arcs where the diameter of these circles corresponds to charge transfer resistances, as shown in Fig. 6d. To have a better analogy, the obtained profiles are fitted to the circuit model shown in the inset of Fig. 6d. In this model, *R*_*s*_ is the series resistance of the cell (including the FTO, electrolyte, etc.), *R*_*ct*_ models the charge transfer dynamics in semiconductor/electrolyte interface, *C*_*ct*_ is the corresponded double layer capacitance, *R*_*sc*_ is the charge transfer resistance in the semiconductor bulk, and *C*_*sc*_ is associated to depletion layer capacitance in the PEC water splitting cell. Moreover, a constant phase element (*Ø*) is employed to model the imperfect capacitance because a pure capacitance is an inaccurate choice for describing the semiconductor/electrolyte solution in actual electrochemical process. According to the fitting findings, the *R*_*ct*_ values for bare TiO_2_, 80 °C TiO_2_-ZnO, and 250 °C TiO_2_-ZnO are found to be 266 Ω, 5.95 Ω, and 15.27 Ω, while the resistance values for *R*_*sc*_ are extracted as 285 Ω, 276 Ω, and 315 Ω, respectively. Based on these findings, the charge transfer dynamics in semiconductor/electrolyte interface have been significantly improved which this further proves the fact that a single ALD cycle of ZnO has passivated trap states in the TiO_2_ NWs surface. Moreover, the similar *R*_*sc*_ resistances for all three cases are an expected feature considering the fact that the modification is in the surface of TiO_2_ NWs, not in its bulk.

## Conclusion

In this study, significant water splitting performance enhancement of TiO_2_ NWs was obtained upon coating with a single ALD ZnO layer at proper deposition temperature. Our findings demonstrate that this angstrom thick layer has positive impact on the activity of TiO_2_ NWs. This improvement is attributed to the defects passivation characteristic of a ZnO layer. As a result of this passivation, the recombination rate of carriers reduces and the density of free carriers is improved. Further improvement in photocatalytic activity of titania NWs can be acquired by choosing the proper deposition temperature. It was found that the layer deposited at a lower temperature has better band alignment and electron transfer dynamics as in the case of 80 °C TiO_2_-ZnO. Originating from all these improvements, the maximum ABPE values are substantiated to 0.23%, and 0.29% for 80 °C, and 250 °C TiO_2_-ZnO samples from the nominal efficiency of 0.2% for bare case. The stability test also reveals that the proposed structure retains its current high under long-term operations. This is mainly due to the ultrathin thickness of ZnO where the layer only passivates the surface traps rather than making a continuous layer. Taking all into account, this study reveals that an angstrom thick layer could significantly improve the photoelectrochemical activity of the photoanode. As the second choice, proper choice of the deposition temperature could substantially intensify this improvement by tuning the interface band alignment in the heterojunction design. The findings of this paper can be utilized in other metal oxide combinations where an angstrom thick passivating layer can lead to higher PEC activity while keeping the long-term stability of the host material intact.

## Electronic supplementary material

Supplementary Information

## References

[1] Fujishima A, Honda K (1972). Electrochemical photolysis of water at a semiconductor electrode. Nature.

[2] Walter MG (2010). Solar water splitting cells. Chem. Rev..

[3] Kudo A, Miseki Y (2009). Heterogeneous photocatalyst materials for water splitting. Chem. Soc. Rev..

[4] Maeda K, Domen K (2010). Photocatalytic water splitting: Recent progress and future challenges. J. Phys. Chem. Lett..

[5] Moniz SJA, Shevlin SA, Martin DJ, Guo Z-X, Tang J (2015). Visible-light driven heterojunction photocatalysts for water splitting – a critical review. Energy Environ. Sci..

[6] Tachibana Y, Vayssieres L, Durrant JR (2012). Artificial photosynthesis for solar water-splitting. Nat. Photonics.

[7] United States. Dept. of Energy. Office of Science. Basic Research Needs for the Hydrogen Economy. Report of the Basic Energy Sciences Workshop on Hydrogen Production, Storage and Use, May 13-15, 2003. *Basic Res. Needs Hydrog. Econ*. 178. 10.2172/899224 (2004)

[8] Ni M, Leung MKH, Leung DYC, Sumathy K (2007). A review and recent developments in photocatalytic water-splitting using TiO_2_for hydrogen production. Renew. Sustain. Energy Rev..

[9] Tang J, Durrant JR, Klug DR (2008). Mechanism of Photocatalytic Water Splitting in TiO_2_. Reaction of Water with Photoholes, Importance of Charge Carrier Dynamics, and Evidence for Four-Hole Chemistry. J. Am. Chem. Soc..

[10] Yin WJ (2010). Band structure engineering of semiconductors for enhanced photoelectrochemical water splitting: The case of TiO_2_. Phys. Rev. B - Condens. Matter Mater. Phys..

[11] Murphy AB (2006). Efficiency of solar water splitting using semiconductor electrodes. Int. J. Hydrogen Energy.

[12] Tian Z (2017). Efficient Charge Separation of *In-Situ* Nb-Doped TiO_2_ Nanowires for Photoelectrochemical Water-splitting. ChemistrySelect.

[13] Altomare M, Lee K, Killian MS, Selli E, Schmuki P (2013). Ta-doped TiO_2_ nanotubes for enhanced solar-light photoelectrochemical water splitting. Chem. - A Eur. J..

[14] Rodríguez-Hernández F, Tranca DC, Martínez-Mesa A, Uranga-Piña L, Seifert G (2016). Water Splitting on Transition Metal Active Sites at TiO_2_-Based Electrodes: A Small Cluster Study. J. Phys. Chem. C.

[15] Wang C (2014). Enhancing visible-light photoelectrochemical water splitting through transition-metal doped TiO_2_ nanorod arrays. J. Mater. Chem. A.

[16] Barakat NAM, Ahmed E, Amen MT, Abdelkareem MA, Farghali AA (2018). N-doped Ni/C/TiO_2_ nanocomposite as effective photocatalyst for water splitting. Mater. Lett..

[17] Xu C, Killmeyer R, Gray ML, Khan SUM (2006). Enhanced carbon doping of n-TiO_2_ thin films for photoelectrochemical water splitting. Electrochem. commun..

[18] Tang J, Cowan AJ, Durrant JR, Klug DR (2011). Mechanism of O_2_ Production from Water Splitting: Nature of Charge Carriers in Nitrogen Doped Nanocrystalline TiO_2_ Films and Factors Limiting O_2_ Production. J. Phys. Chem. C.

[19] Burda C (2003). Enhanced nitrogen doping in TiO_2_ nanoparticles. Nano Lett..

[20] Dholam R, Patel N, Adami M, Miotello A (2009). Hydrogen production by photocatalytic water-splitting using Cr- or Fe-doped TiO_2_ composite thin films photocatalyst. Int. J. Hydrogen Energy.

[21] William BIJr (2002). Efficient Photochemical Water Splitting by a Chemically Modified n-TiO_2_. Efficient Photochemical Water Splitting by a Chemically Modified n-TiO_2_ Ovid: Efficient Photochemical Water Splitting by a Chemically Modified n-TiO_2_. Adv. Sci..

[22] Park JH, Kim S, Bard AJ (2006). Novel Carbon-Doped TiO_2_ Nanotube Arrays with High Aspect Ratios for Efficient Solar Water Splitting. Nano Lett..

[23] Hoang S, Guo S, Hahn NT, Bard AJ, Mullins CB (2012). Visible light driven photoelectrochemical water oxidation on nitrogen-modified TiO_2_ nanowires. Nano Lett..

[24] Xu M, Da P, Wu H, Zhao D, Zheng G (2012). Controlled Sn-doping in TiO_2_ nanowire photoanodes with enhanced photoelectrochemical conversion. Nano Lett..

[25] Yu P, Zhang J (2015). Some interesting properties of black hydrogen-treated TiO_2_ nanowires and their potential application in solar energy conversion. Sci. China Chem..

[26] Hu Yun Hang (2012). A Highly Efficient Photocatalyst-Hydrogenated Black TiO2for the Photocatalytic Splitting of Water. Angewandte Chemie International Edition.

[27] Pesci FM, Wang G, Klug DR, Li Y, Cowan AJ (2013). Efficient suppression of electron-hole recombination in oxygen-deficient hydrogen-treated TiO_2_ nanowires for photoelectrochemical water splitting. J. Phys. Chem. C.

[28] Wang B, Shen S, Mao SS (2017). Black TiO_2_ for solar hydrogen conversion. J. Mater..

[29] Wang D (2014). Photoelectrochemical water splitting with rutile TiO_2_ nanowires array: Synergistic effect of hydrogen treatment and surface modification with anatase nanoparticles. Electrochim. Acta.

[30] Wang G (2011). Hydrogen-Treated TiO_2_ Nanowire Arrays for Photoelectrochemical Water Splitting. Nano Lett..

[31] Wang H (2014). Semiconductor heterojunction photocatalysts: design, construction, and photocatalytic performances. Chem. Soc. Rev..

[32] Liu (2014). 2D ZnIn_2_S_4_ Nanosheet/1D TiO_2_ Nanorod Heterostructure Arrays for Improved Photoelectrochemical Water Splitting. ACS Appl. Mater. Interfaces.

[33] Jang JS, Kim HG, Lee JS (2012). Heterojunction semiconductors: A strategy to develop efficient photocatalytic materials for visible light water splitting. Catal. Today.

[34] Reza Gholipour M, Dinh C-T, Béland F, Do T-O (2015). Nanocomposite heterojunctions as sunlight-driven photocatalysts for hydrogen production from water splitting. Nanoscale.

[35] Choudhary S (2012). Nanostructured bilayered thin films in photoelectrochemical water splitting - A review. Int. J. Hydrogen Energy.

[36] Siripala W, Ivanovskaya A, Jaramillo TF, Baeck SH, McFarland EW (2003). A Cu_2_O/TiO_2_ heterojunction thin film cathode for photoelectrocatalysis. Sol. Energy Mater. Sol. Cells.

[37] Zhou Q, Lin Y, Zhang K, Li M, Tang D (2018). Biosensors and Bioelectronics Reduced graphene oxide/BiFeO_3_ nanohybrids-based signal-on photoelectrochemical sensing system for prostate-speci fi c antigen detection coupling with magnetic micro fl uidic device. Biosens. Bioelectron..

[38] Grądzka I, Gierszewski M, Karolczak J, Ziolek M (2018). Comparison of charge transfer dynamics in polypyridyl ruthenium sensitizers for solar cells and water splitting systems. Phys. Chem. Chem. Phys..

[39] Manfredi N (2018). Dye-Sensitized Photocatalytic Hydrogen Generation: Efficiency Enhancement by Organic Photosensitizer-Coadsorbent Intermolecular Interaction. ACS Energy Lett..

[40] Wang D (2018). Interfacial Deposition of Ru(II) Bipyridine-Dicarboxylate Complexes by Ligand Substitution for Applications in Water Oxidation Catalysis. J. Am. Chem. Soc..

[41] Youngblood WJ, Lee SA, Maeda K, Mallouk TE (2009). Visible Light Water Splitting Using Dye- Sensitized Oxide Semiconductors. Acc. Chem. Res..

[42] Swierk JR, Mallouk TE (2013). Design and development of photoanodes for water-splitting dye-sensitized photoelectrochemical cells. Chem. Soc. Rev..

[43] Shankar Karthik, Mor Gopal K, Prakasam Haripriya E, Yoriya Sorachon, Paulose Maggie, Varghese Oomman K, Grimes Craig A (2007). Highly-ordered TiO2 nanotube arrays up to 220 µm in length: use in water photoelectrolysis and dye-sensitized solar cells. Nanotechnology.

[44] Youngblood WJ (2009). Photoassisted Overall Water Splitting in a Visible Light-Absorbing Dye-Sensitized Photoelectrochemical Cell Photoassisted Overall Water Splitting in a Visible Light-Absorbing. J. Am. Chem. Soc..

[45] Li F (2015). Organic Dye-Sensitized Tandem Photoelectrochemical Cell for Light Driven Total Water Splitting. J. Am. Chem. Soc..

[46] Lee J, Mubeen S, Ji X, Stucky GD, Moskovits M (2012). Plasmonic photoanodes for solar water splitting with visible light. Nano Lett..

[47] Hou W, Cronin SB (2013). A review of surface plasmon resonance-enhanced photocatalysis. Adv. Funct. Mater..

[48] Liu Z, Hou W, Pavaskar P, Aykol M, Cronin SB (2011). Plasmon resonant enhancement of photocatalytic water splitting under visible illumination. Nano Lett..

[49] Xiao, F.-X. & Liu, B. Plasmon-Dictated Photo-Electrochemical Water Splitting for Solar-to-Chemical Energy Conversion: Current Status and FuturePerspectives. *Adv. Mater. Interface*s. **1701098** (2018).

[50] Ghobadi TGU, Ghobadi A, Ozbay E, Karadas F (2018). Strategies for Plasmonic Hot-Electron-Driven Photoelectrochemical Water Splitting. Chem Photo Chem.

[51] Ingram DB, Linic S (2011). Water splitting on composite plasmonic-metal/semiconductor photoelectrodes: Evidence for selective plasmon-induced formation of charge carriers near the semiconductor surface. J. Am. Chem. Soc..

[52] Warren SC, Thimsen E (2012). Plasmonic solar water splitting. Energy Environ. Sci..

[53] Shu J (2018). Plasmonic enhancement coupling with defect-engineered TiO_2_- x: a new mode for sensitive photoelectrochemical biosensing Plasmonic enhancement coupling with defect-engineered TiO_2_-x: a new mode for sensitive photoelectrochemical biosensing. Anal. Chem..

[54] Cai G, Yu Z, Ren R, Tang D (2018). Exciton-Plasmon Interaction between AuNPs/Graphene Nanohybrids and CdS QDs/TiO_2_ for Photoelectrochemical Aptasensing of Prostate-Specific Antigen Exciton-Plasmon Interaction between AuNPs/Graphene Nanohybrids and CdS QDs/TiO_2_ for Photoelectrochem. ACS Sensors.

[55] Xie M (2014). Long-lived, visible-light-excited charge carriers of TiO_2_/BiVO_4_ nanocomposites and their unexpected photoactivity for water splitting. Adv. Energy Mater..

[56] Xie MY (2017). Hydrogen production by photocatalytic water-splitting on Pt-doped TiO_2_–ZnO under visible light. J. Taiwan Inst. Chem. Eng..

[57] Hernández S (2014). Optimization of 1D ZnO@TiO_2_ Core–Shell Nanostructures for Enhanced Photoelectrochemical Water Splitting under Solar Light Illumination. ACS Appl. Mater. Interfaces.

[58] Resasco J (2016). TiO_2_/BiVO4 nanowire heterostructure photoanodes based on type II band alignment. ACS Cent. Sci..

[59] Liu M, Nam CY, Black CT, Kamcev J, Zhang L (2013). Enhancing Water splitting activity and chemical stability of zinc oxide nanowire photoanodes with ultrathin titania shells. J. Phys. Chem. C.

[60] Feng W (2017). Hydrogenated TiO_2_/ZnO heterojunction nanorod arrays with enhanced performance for photoelectrochemical water splitting. Int. J. Hydrogen Energy.

[61] Sarkar A, Singh AK, Khan GG, Sarkar D, Mandal K (2014). TiO_2_/ZnO core/shell nano-heterostructure arrays as photo-electrodes with enhanced visible light photoelectrochemical performance. RSC Adv..

[62] Yun G (2016). Beneficial surface passivation of hydrothermally grown TiO_2_nanowires for solar water oxidation. Appl. Surf. Sci..

[63] Wang W (2016). Heterostructured TiO_2_ Nanorod@Nanobowl Arrays for Efficient Photoelectrochemical Water Splitting. Small.

[64] Pan K (2013). Facile fabrication of hierarchical TiO_2_nanobelt/ZnO nanorod heterogeneous nanostructure: An efficient photoanode for water splitting. ACS Appl. Mater. Interfaces.

[65] Tian J, Hao P, Wei N, Cui H, Liu H (2015). 3D Bi_2_ MoO_6_ Nanosheet/TiO_2_ Nanobelt Heterostructure: Enhanced Photocatalytic Activities and Photoelectochemistry Performance. ACS Catal..

[66] Smith W, Wolcott A, Fitzmorris RC, Zhang JZ, Zhao Y (2011). Quasi-core-shell TiO_2_/WO_3_ and WO_3_/TiO_2_ nanorod arrays fabricated by glancing angle deposition for solar water splitting. J. Mater. Chem..

[67] Zhou W (2010). Ag2O/TiO_2_ nanobelts heterostructure with enhanced ultraviolet and visible photocatalytic activity. ACS Appl. Mater. Interfaces.

[68] Singh AP (2016). Band Edge Engineering in BiVO _4_/TiO_2_ Heterostructure: Enhanced Photoelectrochemical Performance through Improved Charge Transfer. ACS Catal..

[69] Prasittichai C, Hupp JT (2010). Surface modification of SnO_2_ photoelectrodes in dye-sensitized solar cells: Significant improvements in photovoltage via Al2O 3 atomic layer deposition. J. Phys. Chem. Lett..

[70] Ghobadi A (2015). Electrochimica Acta Enhanced Performance of Nanowire-Based All-TiO_2_ Solar Cells using Subnanometer-Thick Atomic Layer Deposited ZnO Embedded Layer. Electrochim. Acta.

[71] Li TC (2009). Surface Passivation of Nanoporous TiO_2_ via Atomic Layer Deposition of ZrO_2_ for Solid-State Dye-Sensitized Solar Cell Applications. J. Phys. Chem. C.

[72] Ulusoy TG, Ghobadi A, Okyay AK (2014). Surface engineered angstrom thick ZnO-sheathed TiO_2_ nanowires as photoanodes for performance enhanced dye-sensitized solar cells. J. Mater. Chem. A.

[73] Chandiran AK (2012). Subnanometer Ga_2_O_3_ tunnelling layer by atomic layer deposition to achieve 1.1 v open-circuit potential in dye-sensitized solar cells. Nano Lett..

[74] Ghobadi A, Ulusoy TG, Garifullin R, Guler MO, Okyay AK (2016). A Heterojunction Design of Single Layer Hole Tunneling ZnO Passivation Wrapping around TiO_2_ Nanowires for Superior Photocatalytic Performance. Sci. Rep..

[75] Ghobadi A (2017). 97 Percent Light Absorption in an Ultrabroadband Frequency Range Utilizing an Ultrathin Metal Layer: Randomly Oriented, Densely Packed Dielectric Nanowires As an Excellent Light Trapping Scaffold. Nanoscale.

[76] Garifullin R (2016). Self-assembled peptide nanofiber templated ALD growth of TiO_2_ and ZnO semiconductor nanonetworks. Phys. Status Solidi Appl. Mater. Sci..

[77] Jacobsson TJ, Edvinsson T (2012). Photoelectrochemical determination of the absolute band edge positions as a function of particle size for ZnO quantum dots. J. Phys. Chem. C.

