# Supplementary material for: Angstrom Thick ZnO Passivation Layer to Improve the Photoelectrochemical Water Splitting Performance of a TiO2 Nanowire Photoanode: The Role of Deposition Temperature
Source: Sci Rep. 2018 Nov 5;8:16322. doi: 10.1038/s41598-018-34248-3 (PMC6218493; doi:10.1038/s41598-018-34248-3)
Supplement: Supplementary file 1 — Supplementary Information [file 41598_2018_34248_MOESM1_ESM.doc]

**Angstrom Thick ZnO Passivation Layer to Improve the Photoelectrochemical Water Splitting Performance of a TiO_2_ Nanowire Photoanode: The Role of Deposition Temperature**

*Amir Ghobadi^a,c,d^, Turkan Gamze Ulusoy Ghobadi^a,b,e^, Ferdi Karadas^a,f*^, Ekmel Ozbay^a,c,d,g*^*

^a^UNAM–National Nanotechnology Research Center, Bilkent University, Ankara 06800, Turkey.

^b^Institute of Materials Science and Nanotechnology, Bilkent University, Ankara 06800, Turkey.

^c^NANOTAM - Nanotechnology Research Center, Bilkent University, Ankara 06800, Turkey.

^d^Department of Electrical and Electronics Engineering, Bilkent University, Ankara 06800, Turkey.

^e^Department of Energy Engineering, Faculty of Engineering, Ankara University, Ankara 06830, Turkey.

^f^Department of Chemistry, Bilkent University, Ankara 06800, Turkey.

^g^ Department of Physics, Bilkent University, Ankara 06800, Turkey.

* Corresponding authors: karadas@fen.bilkent.edu.tr and ozbay@bilkent.edu.tr

**Methods**

***2.1 Materials:*** All of the chemicals were used as received without further purification. Titanium (IV) butoxide (Ti(OBu)4, 97%, Sigma–Aldrich), hydrochloric acid (HCl, 36%, Sigma–Aldrich) and Millipore deionized water (resistivity: 18 mΩ cm) was used for the hydrothermal growth of TiO_2_ NWs on FTO coated glass (2 mm thick, 7 Ω sq^-1^, Solaronix). Diethylzinc ((C_2_H_5_)_2_Zn or DEZn, Sigma-Aldrich) and HPLC-grade water (H_2_O) are used as the zinc and oxygen precursors in the ALD process, respectively.

***2.2 Synthesis of TiO_2_ Nanowires:*** TiO_2_ NW arrays are synthesized by modifying the hydrothermal technique as described in our previous study.^75^ In detail, 1 mL of Ti(OBu)_4_ was added dropwise to the HCl solution, accompanied by intense stirring, which was prepared with the volume ratio of concentrated HCl to DI water being 1:1. After being stirred for 30 min under ambient conditions, the precursor solution was transferred to the a Teﬂon-lined stainless steel autoclave (capacity of 45 mL) with an FTO conducting side facing up with 45° angle against the wall. In advance, FTO glasses are cleaned sufficiently in sonication baths in deionized (DI) water, acetone, and ethanol in sequence, each for 15 min and then dried by N_2_ ﬂow. The hydrothermal reaction was carried out at 180°C for 4 hours, and then it as allowed to cool at the room temperature. Finally, the sample was rinsed with DI water to remove residual solvent, and calcined at 450°C for 2 hours in a tube furnace under air.

***2.3 ZnO Passivation layer of TiO_2_ Nanowires by Atomic Layer Deposition*:** ZnO depositions were carried out at 80°C, and 250°C in an ALD reactor (Cambridge Nanotech Savannah S100).^76^ One cycle of ZnO is applied on TiO_2_ NW arrays as an ultrathin layer with the estimated growth rate of 1.3 Å per cycle. Zn precursor (DEZn) send into the chamber at two different temperatures: 80°C and 250°C. The pulse times of the DEZn and oxygen source (H_2_O) are both 0.015 s, and the purge durations are set at 30 s for both precursors**.**

***2.4 Materials Characterization:*** The morphological characteristics of the synthesized photoanode materials were performed using a scanning electron microscope (SEM, FEI – Quanta 200 FEG) operated at 10 kV. A transmission electron microscope (TEM, Tecnai G2-F30, FEI) was operated at 200 kV. TEM samples were dispersed in ethanol and prepared on a holey carbon coated copper grid. Selected area electron diffraction (SAED) patterns were used to identify the growth direction and crystallinity of the TiO_2_ NWs. X-ray photoelectron spectroscopy (XPS, Thermoscientic K-Alpha, Al K-Alpha radiation, hʋ = 1486.6 eV) measurement has been performed in survey mode by operating a flood gun to prevent surface charging with the pass energy and step size set to 30 eV and 0.1 eV, respectively. XPS is conducted for determining the defect levels and band alignment. To measure the VBM potentials, XPS survey scans were performed to determine the chemical state of ZnO and TiO_2_ and to identify peaks for high resolution analysis with a spot size of 400 μm and a number of scans of 50. Since charge compensation may not be sufficient in eliminating all surface charge, peak positions correction were calibrated by referencing the C1s peak position (284.8 eV) and shifting other peaks in the spectrum accordingly. Thus, charge correction was performed. During the measurements, all samples and electron analyzers were electrically grounded to provide a common reference Fermi level. By using a flood gun, we minimize the differential charging on the surface of oxides during the band offset measurements as well. For the optical characterization of the NWs, a UV-Vis-NIR spectrophotometer (Cary 5000, Varian) was employed. Ellipsometry measurements have also been performed using V-Vase instrument in our desired wavelength regime.

***2.5 Photoelectrochemical measurements:*** Photoelectrochemical measurements were performed using a Gamry Instruments Interface 1000 Potentiostat/Galvanostat in a standard three-electrode electrochemical cell configuration with bare and one cycle ZnO coated (80**°**C or 250**°**C) TiO_2_ NWs with an exposed area of 2 cm^2^, Pt mesh, and Ag|AgCl|KCl(sat) as the working, counter, and reference electrodes, respectively. For the convenience of comparison, all potentials measured vs Ag/AgCl and all of the calculations were based on the geometric surface area. The electrochemical measurements were performed in phosphate-buffered saline (PBS, 0.5 M, pH 7.02) and, prior to each measurement, the electrolyte solution was saturated with N_2_ gas (99.999% purity) for 30 min. The solar light simulator (Sciencetech, Model SLB-300B, 300 W Xe lamp, AM 1.5 global filter) was calibrated to 1 sun (100 mW/cm^2^) using a thermopile optical detector (Newport, Model 818P-010-12). Linear sweep voltammetry (LSV) measurements, transient photocurrent measurements by chronoamperometry technique, electrochemical impedance spectroscopy (EIS) under dark and light conditions, and open-circuit voltage decay (OCPD) were also conducted in the same system. For incident photon-to-current conversion efficiency (IPCE) measurement, a Xenon lamp is utilized as the source. This light is entered into a monochromator (Oriel 1∕8 m cornerstone, 1200 lines∕mm grating) and the output of monochromator is mechanically chopped at 250 Hz and illuminated into the sample. The chopping frequency is fed into a lock-in amplifier (Stanford Research Systems 830) to measure the photogenerated current flowing between the photoanode and the Pt counter electrode. The current density–voltage (*J–V*) curves were measured on TiO_2_-ZnO photoanodes in 0.5M PBS under dark and light conditions with a scanning rate of 10 mV/s between -0.6 and 1V (vs Ag/AgCl) by the LSV technique. To characterize and understand the materials system/interface performance, applied bias photon-to-current efficiency (ABPE) was calculated. EIS were recorded in the frequency range from 10 kHz to 0.01 Hz at open circuit potential (V_OC_) with an alternating current (AC) voltage of 10 mV. Mott-Schottky measurements were performed at potentials varying between -1 and 0.2 **V** (vs Ag/AgCl) to extract electrodes donor densities (*N_d_*) and flat band potentials (*V_fb_*), where the frequency is set at 5 KHz.

Table S1. Atomic concentrations extracted from XPS survey scans

| ZnO deposition temperature, ^o^C | Ti 2p, % | O 1s, % | Zn 2p, % | C 1s, % | Si 2p, % | Zn/(Zn+Ti), % |
| --- | --- | --- | --- | --- | --- | --- |
| 80 | 20 | 50.56 | 7.21 | 21.23 | 1 | 0.265 |
| 250 | 20.61 | 50.15 | 7.17 | 21.36 | 0.71 | 0.260 |

**
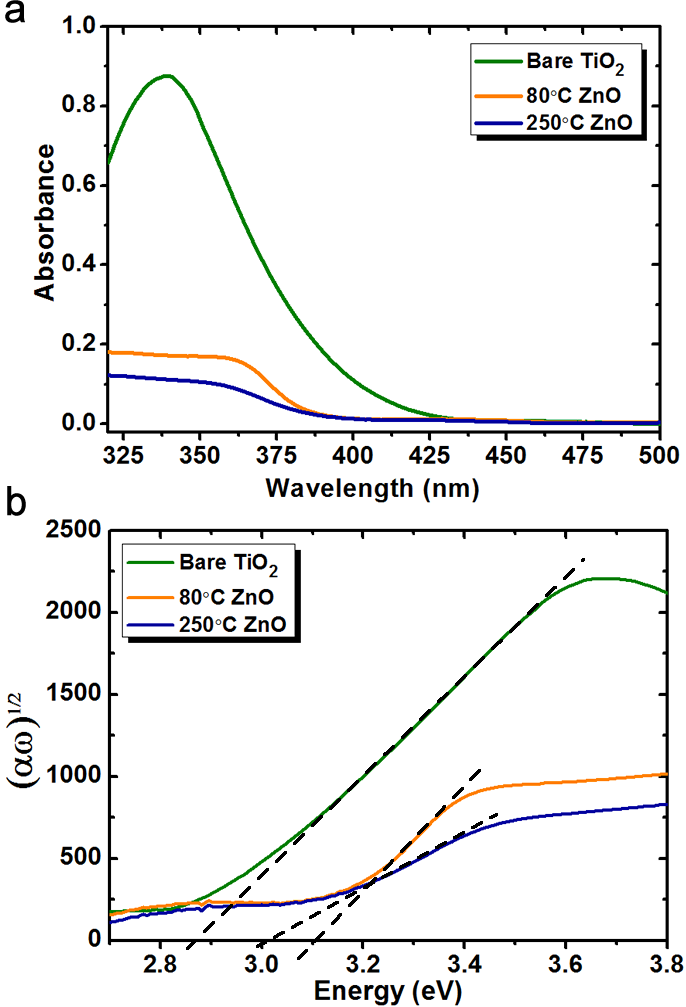
**

**Figure S1.** The bare TiO_2_ NW arrays, 80°C ZnO, and 250°C ZnO bulk samples (a) absorption spectra, (b) Tauc Plots used for the estimation of the optical band gaps (inset).


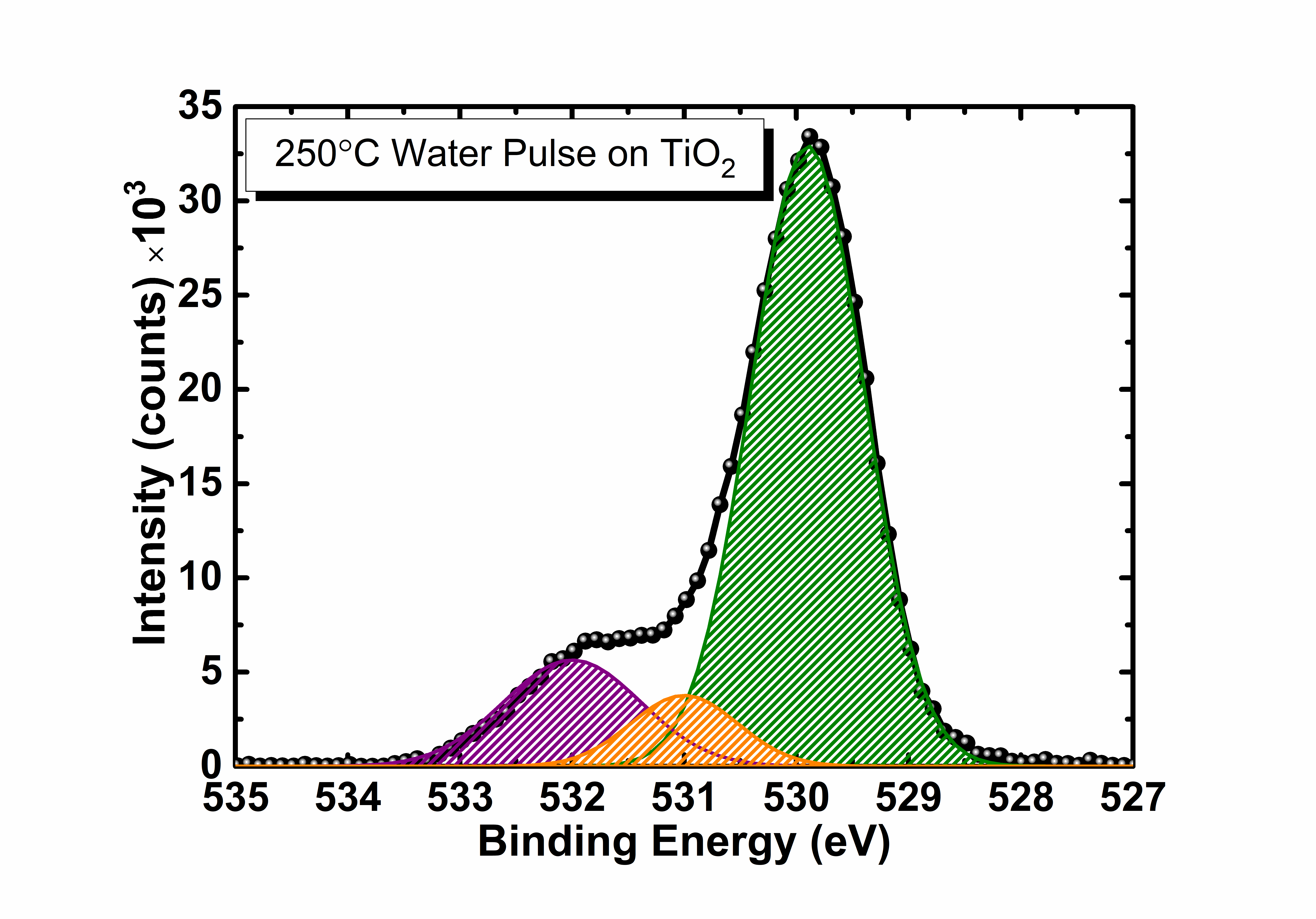


**Figure S2.** The O1s spectra of the TiO_2_ NWs exposed to only water pulse at 250 °C
